# Interpretation of the role of germline and somatic non-coding mutations in cancer: expression and chromatin conformation informed analysis

**DOI:** 10.1186/s13148-022-01342-3

**Published:** 2022-09-28

**Authors:** Michael Pudjihartono, Jo K. Perry, Cris Print, Justin M. O’Sullivan, William Schierding

**Affiliations:** 1 Liggins Institute, The University of Auckland, Auckland, New Zealand; 2 The Maurice Wilkins Centre, The University of Auckland, Auckland, New Zealand; 3 Department of Molecular Medicine and Pathology, School of Medical Sciences, University of Auckland, Auckland, 1142 New Zealand; 4 Australian Parkinson’s Mission, Garvan Institute of Medical Research, Sydney, NSW Australia; 5 MRC Lifecourse Epidemiology Unit, University of Southampton, Southampton, UK

**Keywords:** Cancer, Non-coding mutation, Somatic mutation, Germline mutation, GWAS, eQTL, Chromosome conformation, Hi-C

## Abstract

**Background:**

There has been extensive scrutiny of cancer driving mutations within the exome (especially amino acid altering mutations) as these are more likely to have a clear impact on protein functions, and thus on cell biology. However, this has come at the neglect of systematic identification of regulatory (non-coding) variants, which have recently been identified as putative somatic drivers and key germline risk factors for cancer development. Comprehensive understanding of non-coding mutations requires understanding their role in the disruption of regulatory elements, which then disrupt key biological functions such as gene expression.

**Main body:**

We describe how advancements in sequencing technologies have led to the identification of a large number of non-coding mutations with uncharacterized biological significance. We summarize the strategies that have been developed to interpret and prioritize the biological mechanisms impacted by non-coding mutations, focusing on recent annotation of cancer non-coding variants utilizing chromatin states, eQTLs, and chromatin conformation data.

**Conclusion:**

We believe that a better understanding of how to apply different regulatory data types into the study of non-coding mutations will enhance the discovery of novel mechanisms driving cancer.

## Introduction: the search for germline and somatic variants in cancer has led to an unprecedented generation and sharing of high-quality genomic data

The cells which comprise a malignant tumor carry both germline (inherited) and somatic (acquired) genetic variants within their genome, some of which may be pathogenic. Germline variants are inherited from an individual’s parents and therefore are present in every cell, not just malignant cells. A subset of these germline variants affects cellular mechanisms that alter an individual’s lifetime risk (predisposition) of developing cancer [1]. In contrast, somatic mutations accumulate throughout an individual’s lifetime and are acquired de novo by each cell through exposure to various endogenous and exogenous factors [2]. Importantly, a subset of somatic mutations alters cellular mechanisms in such a way as to grant cells an increased ability to survive and/or proliferate, which is one of the hallmarks of cancer [3]. There is a greater likelihood that cells that harbor the right set of these “advantageous” germline and somatic mutations will be positively selected and undergo tumorigenesis. However, not every mutation is implicated in tumor development. Overall, the typical tumor contains two to eight such “advantageous” mutations, with all remaining mutations as passengers that confer no selective growth advantage [4]. Therefore, identifying key cancer-associated germline and somatic variants has been the primary goal for many past and present cancer studies, putting together patterns of mutational signatures into clues that infer ideal treatment strategies.

Heritable cancer risk genes were initially discovered in the 1980s and 1990s through genetic linkage studies in families with a clear tumor inheritance pattern. Within these genes, early mutations act as dominant Mendelian mutations, where a single mutant copy of the disease-associated gene is enough to confer cancer risk. These early studies identified high-penetrance susceptibility genes for breast cancer (*BRCA1* and *BRCA2*) [5–7], colorectal cancer (*APC*, *MLH1*, *MSH2*) [8–12] and melanoma (*CDKN2A*) [13–15]. However, mutations in these high-penetrance genes only account for a small fraction of the total heritability of their respective cancer types [16–18]. For example, less than 25% of breast cancer inheritance is due to known high-penetrance genes (including *BRCA1* and *BRCA2*) [19]. This leaves much cancer heritability to be explained by the combined effect of many low-penetrance germline variants (polygenic inheritance model) [20]. Unfortunately, while linkage study is appropriate for identifying high-penetrance genes like *BRCA1* and *BRCA2*, it lacks the power to detect low-penetrance alleles [21]. Thus, methods beyond linkage analysis are needed to identify polygenic germline susceptibility variants.

Technical limitations also hampered the early identification of somatic mutations linked to cancer. Despite this, low-throughput techniques such as targeted Sanger-based sequencing and cytogenetics have successfully identified many recurrent somatic mutations [4, 22, 23] and have led to the development of successful targeted therapies [24, 25]. However, these early methodologies were nonetheless limited by cost and throughput: only a limited number of genes can be analyzed, and these genes must be targeted a priori. From 2005 onward, advancements in genotyping and next-generation sequencing technologies accelerated the search for germline and somatic variants in cancer. For germline mutations, the ability to conduct large case–control studies (i.e., genome-wide association studies; GWAS) to systematically assay millions of common genetic variants across hundreds of thousands of individuals led to the discovery of hundreds of new susceptibility loci for many cancer types [26]. Similarly, high-throughput DNA sequencing revolutionized the identification of somatic mutations by enabling the sequencing of normal versus tumor exomes [27–31] and whole genomes [32–35]. For both germline and somatic variants, large collaborations, including the Cancer Genome Atlas (TCGA) [36] and the International Cancer Genome Consortium (ICGC) [37], have facilitated the sequencing and sharing of thousands of normal and tumor genomes. This unprecedented data access has further accelerated the discovery and analysis of malignancy-driving mutations by enabling individual labs to access tumor genomic data without the need to perform sequencing.

## The misunderstanding of the non-coding genome as merely passenger events has led to a gap in functional interpretation

Despite the success of variant identification over the past two decades, there is still a sizeable gap in our understanding of how germline variants influence cancer susceptibility. Arguably, one of the biggest contributing factors to this knowledge gap is the finding that > 90% of identified GWAS variants lie in the non-coding regions of the genome [38], making their direct functional interpretation difficult.

Similarly, most somatic variants identified through whole-genome sequencing of tumor samples lie outside of known protein-coding regions [39]. Due to the lack of a causative change in protein structure, non-coding somatic variations are traditionally seen as neutral or “passenger” events (as opposed to “driver”), with no function in driving tumorigenesis. However, recent findings have challenged this view and have highlighted the importance of non-coding aberrations in driving tumorigenesis through the targeting of a diverse set of functional elements [40–45].

The most characterized somatic non-coding mutation in human cancer is the *TERT* (telomerase reverse transcriptase) promoter, which is recurrently mutated in more than 50 individual cancer types [46]. In melanoma, mutations in the *TERT* promoter occur in ~ 80% of cases [40] and are associated with poor patient outcome [47]. *TERT* promoter mutations drive carcinogenesis by creating de novo binding sites for ETS (E26 transformation-specific) transcription factors, leading to increased transcription of the catalytic subunit *TERT* [48, 49]. In turn, this activates the telomerase complex, which is normally deactivated in somatic cells. The reconstitution of telomerase activity enables cells to maintain telomere length and thus escape telomere-initiated cellular senescence. As a consequence, the mutated cells can divide and proliferate indefinitely, one of the hallmarks of cancer [3].

Recurrent non-coding mutations have also been identified in enhancer sequences 4 kb upstream of the transcriptional start site of the *LMO1* oncogene in T cell acute lymphoblastic leukemia [50]. These mutations generate a new binding site for the MYB transcription factor, enhancing expression of *LMO1* [50].

Despite their abundance, few other non-coding mutations have had such clear interpretations of their biological consequences. As such, there has been an increasing interest in the identification and interpretation of non-coding variants in cancer. For example, the Pan-Cancer Analysis of Whole Genomes (PCAWG) has recently conducted an ambitious re-analysis of ICGC and TCGA whole-genome sequencing (WGS) data from more than 2600 cancer patients across 38 different primary tumors [51]. This resulted in the discovery of novel non-coding driver mutations in 25% of tumor samples, with one third of those affecting the *TERT* promoter (237 of 785). Additional identified drivers include non-coding point mutational hotspots near *TP53*, *TOB1*, *NFKB1Z*, and the *RMRP* promoter [44]. However, the vast majority of these non-coding modifications result in loss of function, which is inherently more difficult to therapeutically target than gain of function. Thus, better molecular understanding is required to identify treatments which interfere with these adaptive processes, such as targeting of germline non-coding variants as both a preventive and a therapeutic strategy.

## Strategies for resolving the gap in functional interpretation of cancer variants

### Mutation prioritization strategies

Previously referred to as “junk” [52], the non-coding genome is now recognized as containing a large number of functional elements known as *cis*-regulatory elements (CREs) [53]. CREs are functional elements within the non-coding genome that can regulate the transcription of genes. The main types of CREs include promoters and enhancers [54]. Due to their role in regulating gene expression, CREs provide discrete intervals in which to search for functionally important mutations. Thus, the most straightforward way of gaining functional insight is by overlapping non-coding mutational data with known CREs. This approach prioritizes mutations that are most likely to have a functional effect and thus infers a likely biological function of the non-coding mutations (“mutation prioritization”).

**Fig. 1:**
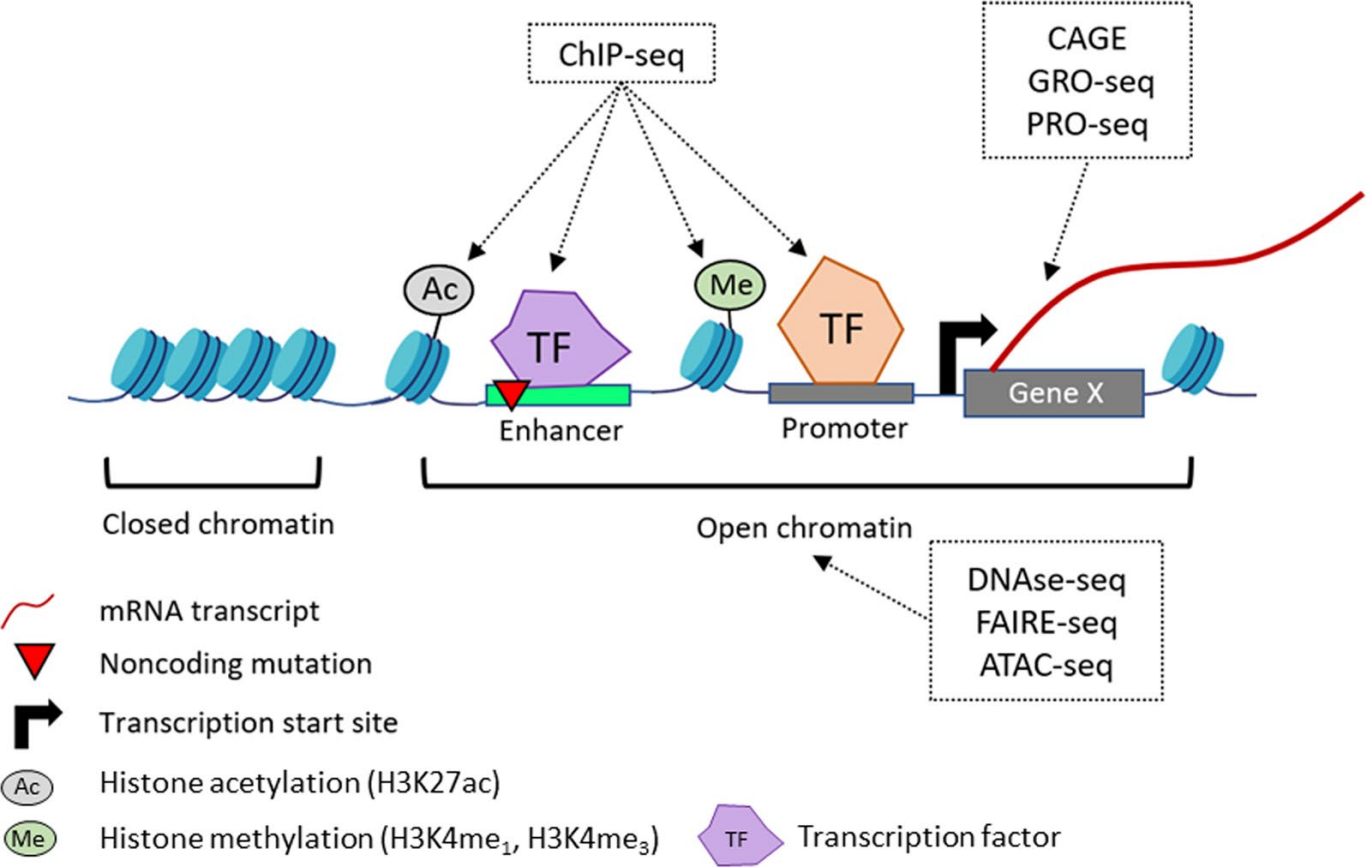
An overview of *cis*-regulatory elements and the experimental methods to identify them

Many experimental methods are available to identify putative CREs in a given tissue or cell type (Fig. 1). These methods typically exploit different features of active CREs. For example, active regulatory elements are known to reside in open chromatin regions to allow for transcription factor binding. As such, methods that detect open chromatin regions (e.g., DNase-seq [55], FAIRE-seq [56], and ATAC-seq [57]) or transcription factor binding (ChIP-seq [58]) can be used as a proxy to identify active regions, which are a necessary condition for identification of putative active CREs. In addition, active enhancer regions are marked by a specific combination of histone modifications (e.g., H3K27ac, H3K4me_1_, and H3K4me_3_ [59]), which can be detected using ChIP-seq. Finally, methods that capture transcriptional activity such as CAGE [60], GRO-seq [61], and PRO-seq [62] can quantify the transcription of genes and enhancers to identify transcriptionally active regions. Vast volumes of such genome-annotation datasets across many different cell types are available through public databases such as the NIH Roadmap consortium [63], IHEC consortium [64], ENCODE [53], and FANTOM5 [65]. With the availability of so many different types of annotation data, computational tools can combine annotation data from different databases to intersect non-coding variants with identified regulatory elements. Examples of such tools include Ensembl Variant Effect Predictor [66] and FunciSNP [67]. Importantly, each of these tools uses a different subset of available annotation data and thus may come to a different conclusion as to which mutations should be prioritized for follow-up. Recent tools such as GWAVA [68], DeepSEA [69], and Sei [70] use machine learning classification models to prioritize non-coding mutations. For example, based on a modified random forest algorithm, GWAVA prioritized five SNPs inside the 3’UTR of the caveolin 2 gene, *CAV2* [71]. Through further investigations, one of the SNPs (rs10249656) was found to abolish an miRNA (miR-548s) binding site, leading to increased *CAV2* expression, thus providing a plausible explanation for its association with pancreatic cancer [71]. However, comparison across different machine learning models can become problematic since these tools uses different datasets to train their algorithms, which can affect the prioritized variants [72]. Overall, there is currently no consensus as to which prioritization tools are best.

### Gene prioritization strategies

While informative, mutation prioritization strategies which rely on identification of regulatory elements only identify the putative ability of a genetic variant/mutation to dysregulate gene expression. To fully elucidate the underlying mechanism of its involvement in tumorigenesis, the next step is to identify the transcripts that are affected by this disruption. This task is much more challenging for enhancers as, unlike promoters which are typically located immediately upstream of their target gene [73], enhancers can be located upstream, downstream, within the intron of a gene, or even thousands of base pairs away [74].

**Fig. 2:**
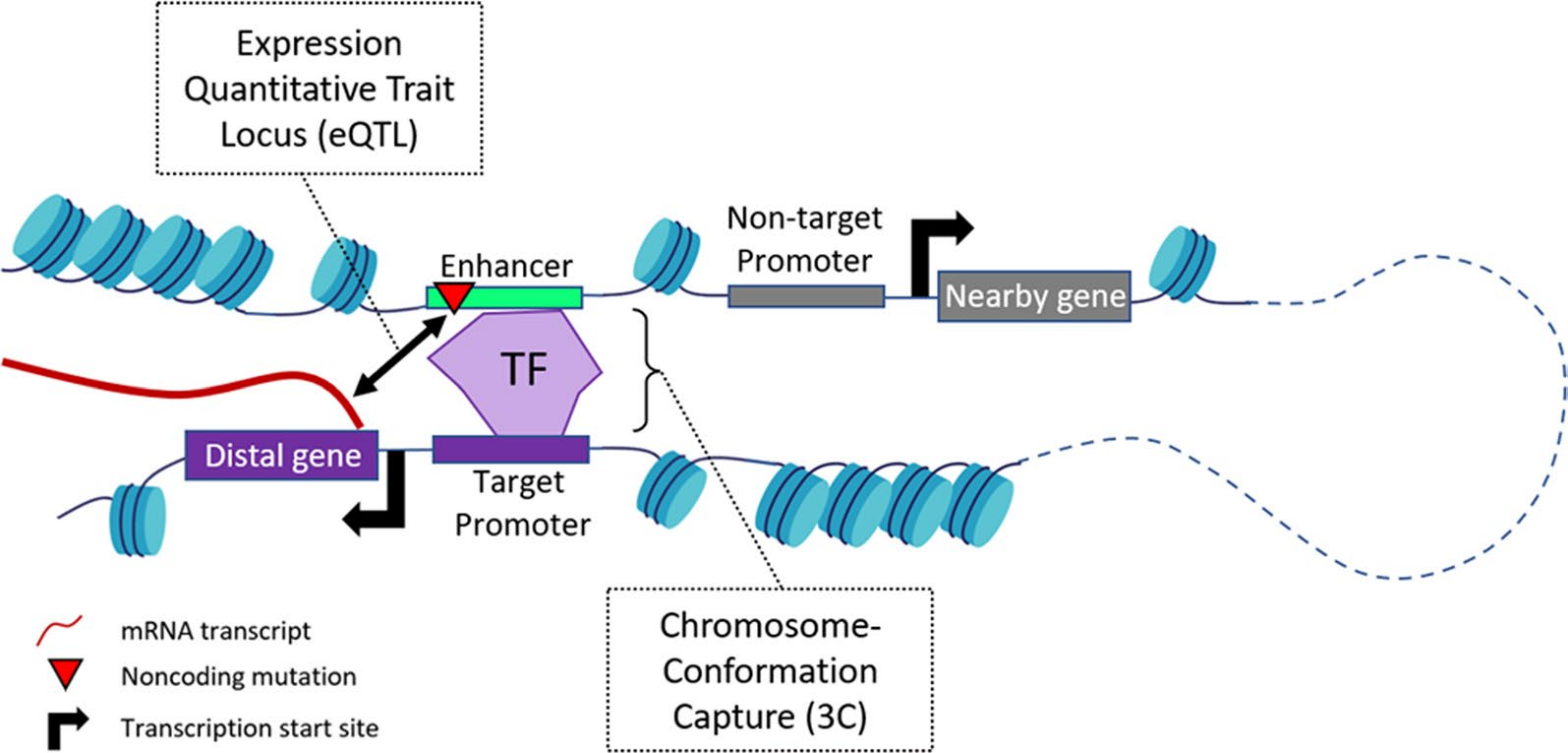
Methods to link enhancers to their target genes

Several methods are available to prioritize candidate genes in order of their potential to be targeted by an enhancer mutation. Such “gene prioritization” methods can include a multitude of data types, but current tools are largely confined to: (1) nearest gene, usually based on correlation to coding mutations using linkage disequilibrium (proximity-based association); (2) nearest gene on the basis of prior knowledge about the biological function (functional association); (3) target gene on the basis of a statistical association between the mutation and gene expression levels (expression quantitative trait loci; eQTL); or (4) target gene based on physical looping of the mutated region to a gene promoter (chromosome conformation capture; 3C). The fundamental limitation with the first two strategies is that enhancers do not necessarily target the nearest genes but can bypass neighboring genes to regulate genes located further away on the linear genome [75] (Fig. 2). As such, assigning target genes based on linear proximity is not ideal and can lead to false assignments. This is exemplified by studies of obesity and body mass index GWAS variants that are located at the intron of *FTO*. Due to its linear proximity, *FTO* was initially thought to be the target gene of these regulatory variants [76, 77]. However, expression level, chromosome conformation, and other experimental evidence later indicated that *IRX3*, a distal gene, was the likely target gene [78, 79].

Many computational tools can be used to aid in gene prioritization. These tools usually incorporate additional data sources in the form of eQTL data (e.g., eCAVIAR [80], RegulomeDB [81], HaploReg [82], CADD [83], ANNOVAR [84], Sherlock [85], coloc [86], GPRM [87], and PINES [88]), chromosome conformation data (e.g., GWAS3D [89], H-MAGMA [90]), or both (CoDeS3D [91], FUMA [92]) to arrive on potential target genes in addition to providing functional annotation. As with the mutational prioritization tools, the varying annotation datasets used by each gene prioritization tool means that these tools often do not agree with each other. These inconsistencies have been addressed by tools that use machine learning to combine features/scores from multiple tools into a single score for easier interpretation and benchmarking. For example, SURF [93] combines features from RegulomeDB and DeepSEA to predict the effect of regulatory variants on gene expression using a random forest algorithm.

Taken together, eQTL and chromosome conformation are powerful resources that can help to resolve the gap in functional interpretation by linking non-coding variants to their target genes. The following sections will discuss these concepts in more detail.

## Leveraging expression quantitative trait loci (eQTL) associations to identify the target genes of non-coding variants

Intermediate phenotypes lie between genetic variation and disease. The expression level of a protein-coding gene is an intermediate phenotype that may be responsible for mediating the connection between a non-coding genetic variant and its association with disease susceptibility [94]. Therefore, understanding the relationship between non-coding genetic variants and gene expression levels may shed light into the mechanisms that drive tumorigenesis.

**Fig. 3:**
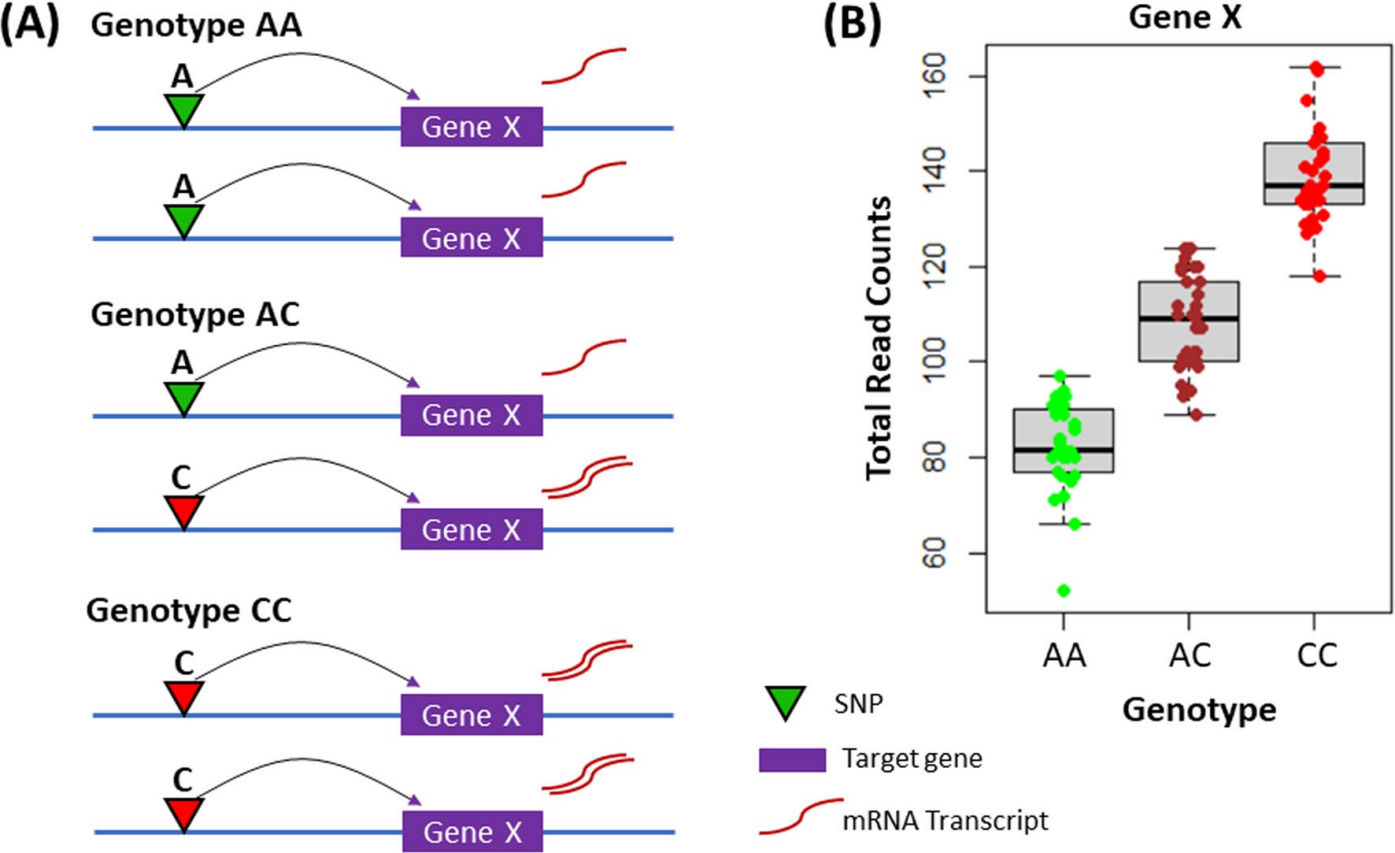
Expression quantitative trait locus. **A** An expression QTL arises due to DNA variation (single nucleotide polymorphism; SNP) modifying the transcription level of target gene X in an allele-specific manner. **B** Association between a SNP’s genotype and the expression level of target gene X in hundreds of individuals

An expression quantitative trait locus (eQTL) is a genetic locus (usually marked by single nucleotide polymorphism; SNP) where genotype associates with a fraction of the variability of a gene (or transcript) expression phenotype [95] (Fig. 3A). Thus, to find eQTLs, two sources of information are needed: genotype and matched gene expression data. Using these datasets, it is possible to perform association tests between each SNP-gene pair in many individuals by regressing the number of alternative alleles versus gene expression using a linear model (where significance of the slope is the significance of the eQTL) (Fig. 3B). Therefore, significant eQTLs identify a target gene and can lead to better functional interpretation of the mechanism underlying a significant SNP-disease association. For example, using pan-cancer, donor-matched expression data, an eQTL between non-coding SNP rs2142833 and *APOBEC3B* expression levels (*β* = 0.19, *P* = 2 × 10^−6^) confirmed germline risk as arising from alteration of expression within the APOBEC3 family of cytidine deaminases [51].

### Tissue and cell-type specificity of eQTLs

Early eQTL mapping studies mainly focused on finding eQTLs in whole blood or blood-derived cells due to sample accessibility [96, 97]. However, subsequent comparative studies have revealed that eQTLs can be highly tissue specific [98–102]. For example, a comparison between cortical tissue and peripheral blood mononucleated cells showed less than 50% overlap in regulatory associations [100]. In addition, recent evidence points to blood eQTLs having a weak correlation with the eQTLs discovered in other tissues, especially neural [101]. Therefore, a genetic variant may be an eQTL to a particular target gene in one tissue but not in other tissues. Thus, it is imperative that the eQTL data be matched to the tissue or organ relevant to the disease state, something available in publicly available databases such as the genotype-tissue expression (GTEx), which contains eQTLs from hundreds of individuals across 54 healthy human tissue types [102].

Beyond tissue specificity, capturing cell-type-specific eQTLs requires going beyond bulk tissue samples [103–109]. Identifying cell-type-specific eQTLs (ct-eQTL) and single-cell eQTLs (sc-eQTL) requires cell-type isolation or single-cell RNA-seq across thousands of cells per individual, such as that generated by Fairfex et al. for B cells and monocytes [108]. Indeed, bulk approaches can be less effective if the tissue of interest is composed of highly heterogeneous cell types [110]. This is especially relevant for melanoma, which arises from melanocytes: a cell type that typically accounts for less than 5% of cells captured by human skin biopsies. Recently, the first melanocyte-specific eQTL dataset was published by Zhang et al [109]. Through ct-eQTL analysis, Zhang and colleagues were able to identify melanocyte-specific regulation between SNPs in five known melanoma GWAS loci and their target driving genes [109]. For example, *PARP1* was identified as the target gene regulated by the melanoma-associated locus 1q42.12, agreeing with previous reports of *PARP1* acting as a melanoma susceptibility gene in a melanocyte lineage-specific manner [111]. Similarly, *SLC45A2*, a gene known to be involved in the melanin synthesis pathway [112], was also prioritized through ct-eQTL analysis. Importantly, these associations could not be captured using the two available GTEx bulk skin datasets, thus highlighting the value of ct-eQTL analysis in capturing associations that would otherwise be masked using bulk approaches [109].

### Leveraging eQTL datasets to prioritize functional genes at GWAS loci through gene-based association testing

**Fig. 4:**
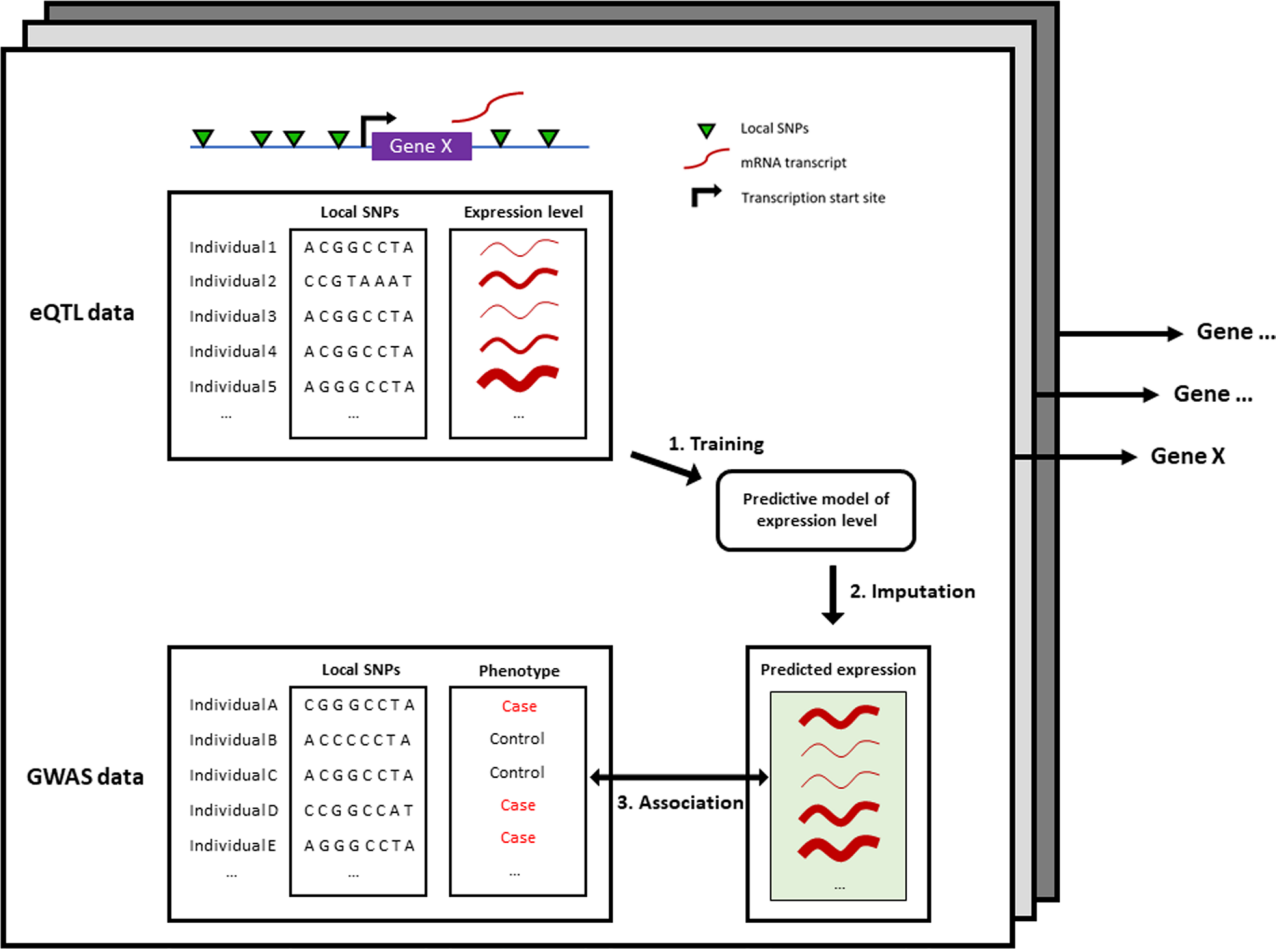
An overview of TWAS pipeline (PrediXcan [116]). The general method of TWAS is composed of three steps. First, using individual-level genotype and matching gene expression data from a reference eQTL dataset, predictive models are trained to estimate the expression level of each gene based on local genotype. Second, the models are used to predict (or “impute”) the expression level of genes (normally not captured in GWAS) for each individual-level genotype in a GWAS dataset. Third, an association test is conducted for each predicted expression with the trait to elucidate gene–trait associations. The first step has been improved by subsequent methods, which allow summary-level GWAS data as input (e.g., FUSION [117], S-PrediXcan [118], MOSTWAS [119], and UTMOST [120]). For example, UTMOST takes summary-level data and simultaneously trains models across multiple tissues to increase power [120]

Leveraging the growing number of eQTL datasets (e.g., GTEx [102], GEUVADIS [113], DGN [114], and Braineac [115]), transcriptome-wide association studies (TWAS) identify the gene–trait associations underlying GWAS variant–trait associations [116] (Fig. 4). TWAS hypothesize that the expression level of each gene is modulated by one or multiple eQTLs, and that the genetically altered expression level of genes underlies specific traits (i.e., disease risk). For example, using melanocyte ct-eQTL data as a reference dataset, TWAS allowed the prioritization of genes at three known melanoma GWAS susceptibility loci [109].

Due to the nature of TWAS, which combines the effect of multiple regulatory variants into a single testing unit (a gene), an increase in power is achieved compared to traditional GWAS. For example, using melanocyte ct-eQTL data, TWAS also successfully prioritized five genes at four novel melanoma susceptibility loci, which were later verified as genome-wide significant in a larger and more recent melanoma GWAS meta-analysis [121] or melanoma and nevus count pleiotropic analysis [122]. As such, TWAS can nominate not only functional genes at known GWAS loci but also discover new loci previously unidentified by GWAS.

As with standard eQTL analysis, the use of non-trait-relevant tissues/cell types can introduce bias. However, using slightly less related tissues in TWAS to considerably increase sample size was shown in melanoma (using three non-melanocyte tissues: GTEx sun-exposed and not sun-exposed skin and transformed skin fibroblast) to successfully identify a novel melanoma susceptibility locus [121]. While the use of melanocyte-specific data still yields better results (identified six novel loci), using non-melanocyte data supplemented the findings of melanocyte data [121]. Overall, the trade-off between tissue bias and information loss due to smaller sample size should be evaluated on a case-to-case basis [123].

### Genomic clumping to detect somatic eQTLs

Unlike germline SNPs, the number of somatic mutations occurring at the same genomic location across a study population is expected to be low [124]. Therefore, to infer a correlation between a non-coding somatic mutation and gene expression level (somatic eQTLs), researchers take a collapsing strategy whereby nearby variants are grouped together into a single “locus” for burden testing. This technique has the advantage of increasing the effective mutation minor allele count and, thereby, increasing statistical power. This merging is effective because multiple alterations from different genomic locations can consistently affect regulation of a particular gene [41]. For example, somatic single nucleotide variants (SNVs) from 930 TCGA tumor samples within 50 bp of each other were grouped together to define recurrently mutated loci that could act as somatic eQTLs [125]. This identified somatic eQTLs frequently mutated in melanoma, including 12 that were almost exclusively mutated in melanoma, and two loci that regulate the expression of *DAAM1* (191 bp downstream) and *HYI* (95 kb away) [125].

DAAM1 is a protein that plays a vital role in the recruitment of actin cytoskeleton and is thought to contribute to cancer invasiveness by increasing cell motility [126–128]. The *HYI* somatic eQTL was proposed to associate with increased *HYI* expression by altering an ETS binding motif [125]. *HYI* encodes a hydroxypyruvate isomerase [129] and thus may contribute to cancer by affecting the transport and metabolism of carbohydrates. These associations were confirmed through experimental validation, indicating a causal relationship [125]. Thus, through genomic clustering, non-coding mutations were attributed to alteration of melanoma-relevant gene expression in several important gene loci.

### Considerations for somatic and germline eQTLs

Both germline and somatic eQTLs have specific weaknesses in identifying functional non-coding mutations. Like GWAS, the study of germline eQTLs is complicated by population-based study weaknesses such as co-inheritance and population stratification. There is a strong tendency of nearby SNPs to be co-inherited, leading to blocks of genomic variants inherited together across a population (in strong linkage disequilibrium; LD). If a genomic region contains multiple co-inherited variants, then variants in strong LD will be indistinguishable between marker variants and the variant truly causative of the gene expression changes (causal variant). To address this, fine-mapping approaches can disentangle the causal variant from those merely in LD with it. For example, CAVIAR [130], CAVIARBF [131], FINEMAP144 [132], CaVEMaN145 [133], and SuSiE146 [134] use a Bayesian approach to elucidate a “credible set” of variants containing the true causal variant with high probability (e.g., 95%). An extension of CAVIAR, called eCAVIAR80 [80], is a gene prioritization tool that uses the same Bayesian principle to estimate the probability of the same GWAS and eQTL variants being causal given the uncertainty of LD. This type of gene prioritization approach, leveraging two data types together, is called colocalization. For example, Zhang et al. [109] used eCAVIAR in their ct-eQTL analysis to find the causal eQTL variants that colocalize with melanoma GWAS signals to identify the likely functional genes on the two GWAS loci (*PARP1* and *SLC45A2*).

In contrast, somatic variants are not inherited and thus, by definition, arise independently from each other. Therefore, controlling for LD is not a concern in somatic eQTL analysis. However, identification of somatic eQTLs is challenging due to the dependence on the availability of tumor and matched normal samples. The use of cancer samples as a control set is unfavorable since other cancer events can influence the expression of target genes. Thus, paired statistical tests between tumor and matched normal samples are required to detect significant associations. Secondly, somatic variants arise de novo, meaning that a comprehensive method like whole-genome sequencing is needed to identify them. This contrasts with common germline variants that can be catalogued and put into SNP arrays, making their identification considerably cheaper.

## The spatial organization of the genome as a tool to further explain the functional target of non-coding variants

**Fig. 5:**
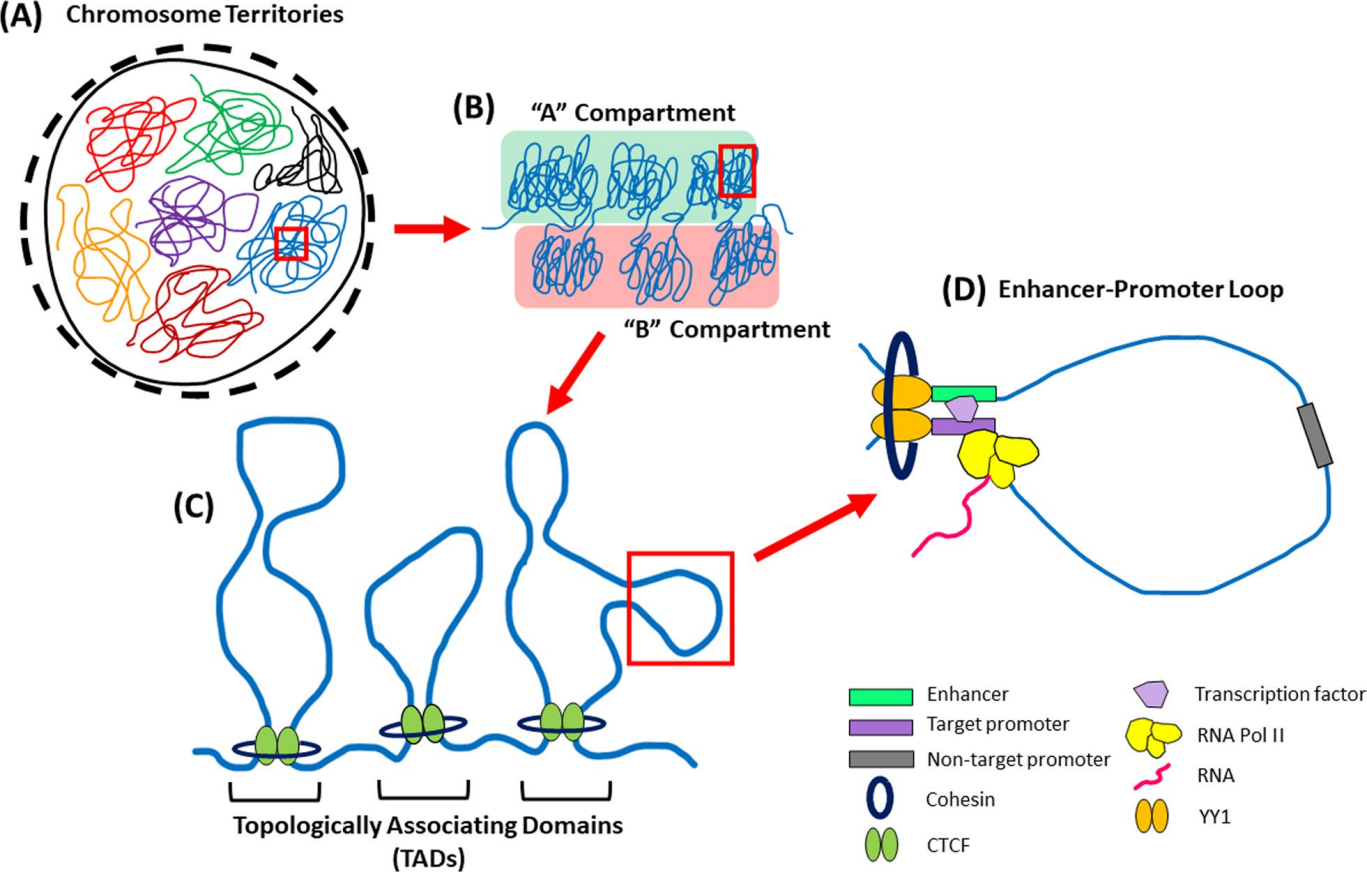
The non-random packaging of chromatin inside the nucleus. **A** On the nuclear scale, each chromosome occupies individual regions, termed chromosomal territories [135]. **B** Within these chromosomes, chromatin clusters into transcriptionally active (“A”) and inactive (“B”) compartments [136]. **C** Within these compartments, further organization occurs in the form of megabase-long loop structures called “topologically associating domains” (TADs) [137]. TADs are highly conserved between cell types and tend to insulate enhancers and genes contained within it from elements outside of the TAD, thereby preventing inappropriate enhancer–promoter contacts [138]. **D** Finally, TADs are further compartmentalized into smaller sub-TAD loops that frequently facilitate enhancer–promoter interactions. Unlike TADs, these smaller loops are more cell-type specific [139]. Generally, it is thought that TAD and sub-TAD loops are formed by the interaction of CTCF DNA binding proteins and cohesin ring-shaped complexes that bring distant chromatin regions into physical proximity [140]. However, even this is a simplistic model as further evidence suggest the involvement of many other factors. For example, recent evidence suggest that sub-TAD loops are more commonly stabilized by YY1 proteins in a manner analogous to CTCF [141]. Another evidence shows the involvement of ZNF143 as a chromatin-looping factor that bind to promoter and establish loops through interaction with enhancer-bound CTCF and cohesin [142]. Overall, the mechanisms behind chromatin looping are still an active area of research

One way gene expression is regulated is through the formation of physical loops that connect distal regulatory elements (e.g., enhancers) to the promoters of their target genes, resulting in the recruitment of transcription factors/cofactors that activate transcription from the target promoters [143]. Importantly, this mechanism of regulation is directly linked to the three-dimensional organization of the genome. Within each cell, DNA fits inside the nucleus through the systematic packaging of chromatin into an exquisite hierarchical structure (Fig. 5A–C). Within this structure, regions of DNA are further compartmentalized into chromatin loops that connect regulatory elements with their target gene promoters (Fig. 5D). These enhancer–promoter loops are cell-type specific, which contributes to tissue-specific gene regulation [139]. To capture the connections formed by three-dimensional chromatin folding, methodologies such as chromosome conformation capture (3C) [144] and its derivatives (e.g., 4C [145, 146], 5C [147], GCC [148], and Hi-C [136]) have been developed. Overall, Hi-C is the most extensive examination, enabling the elucidation of the physical interaction of all genomic loci in an unbiased manner (all vs. all). Importantly, such methodologies can be leveraged to identify enhancer–promoter loops, thus facilitating the identification of target genes [149].

The DNA–protein complexes that prevent inappropriate enhancer–promoter contacts are frequently mutated in cancer. For example, somatic mutations in the eight genes that comprise the cohesin ring (*SMC1A*, *SMC3*, *STAG1*, *STAG2*, *RAD21*) and the cohesin-ring support genes (*NIPBL*, *MAU2*, *WAPL*, *PDS5A*, *PDS5B*) and *CTCF* are frequently found in many cancer types [150] and are especially common in acute myeloid leukemia (AML) [151–153]. In AML, cohesin subunit knockdown has been shown to alter gene transcription, likely through the disruption of cis-regulatory architecture [154, 155]. Thus, cohesin mutations likely drive tumorigenesis by altering the three-dimensional genome organization, resulting in aberrant gene expression [152]. Across all cancer types, the mutation rate of *CTCF* is 2% overall, with the mutation considered to be oncogenic in half the cases [51]. Mutations in cohesin/CTCF binding sites are also frequently found in cancers, altering regulatory interactions in AML (activating *TAL1* [156]), melanoma, and gastric cancer [45]. Abnormal expression of *ZNF143* is related to a wide range of pathogenic behaviors in cancer cells [157]. Additionally, depletion of YY1 or deletion of its binding sites have been shown to disrupt normal gene expression [141]. Thus, understanding genome organization and the specific connections between two genomic locations can be leveraged to describe one type of regulatory mechanism modulating key biological functions in cancer.

Beyond direct mutation of the structural machinery, it has been shown that many disease-associated non-coding mutations alter regulatory elements involved in chromatin organization and looping [158, 159]. The use of Hi-C data to elucidate the target genes of these non-coding variants has allowed for functional interpretation of many germline cancer-associated loci, including breast cancer [160], colorectal cancer [161, 162], prostate cancer [163, 164], pancreatic cancer [165], papillary thyroid carcinoma [166], and melanoma [167] [discussed below].

### Functional interpretation of the germline melanoma risk locus 7p21.1

The melanoma risk locus 7p21.1 represents an interesting case study, as initial efforts to interpret its biological mechanism yielded inconclusive results. This locus was first identified through a GWAS meta-analysis in 2015 with rs1636744, which is 63 kb from *AGR3*, identified as the most significant variant in the locus [168]. However, the region surrounding rs1636744 was not conserved between primates, suggesting little functional significance [168]. Furthermore, while rs1636744 and two other SNPs within this locus (rs847377 and rs847404) are eQTLs for *AGR3* in GTEx lung tissue, they are not eQTLs in sun-exposed skin. In 2018, the nearby rs117132860 variant was associated with decreased tanning ability [169]. This suggests that variants at 7p21.1 might act on melanoma disease risk through the modulation of tanning response. In 2020, the most significant melanoma association was adjusted to rs117132860123, which was also the lead signal for association with cutaneous squamous cell carcinoma [170]. However, the function behind these associations remained elusive.

By using a targeted Hi-C approach, a recent 2021 study in primary melanocytes was able to infer a physical association between the region containing rs117132860 and the promoter of *AHR* [167]. Using ATAC-seq, ChIP-seq, and DNase-seq, rs117132860 was shown to lie in an open chromatin region marked by enhancer activity and located within an *AHR* binding motif. Furthermore, eQTL analysis using a melanocyte-specific dataset [109] showed a strong correlation between the A-risk allele and lower *AHR* expression180 [167]. As *AHR* plays an important role in the cellular response to dioxin and UV radiation183–186 [171–174], together these data suggest that rs117132860 is a causal variant within a UV-responsive element that confers disease risk through the modulation of *AHR* expression. Together, this evidence suggests that this locus has a gene–environment interaction whereby UV radiation interacts with the at-risk genotype as a basis for the association in this locus to melanoma, tanning response, and cutaneous squamous cell carcinoma.

### Chromosome conformation decodes gene-level recurrence for non-coding somatic mutations

**Fig. 6:**
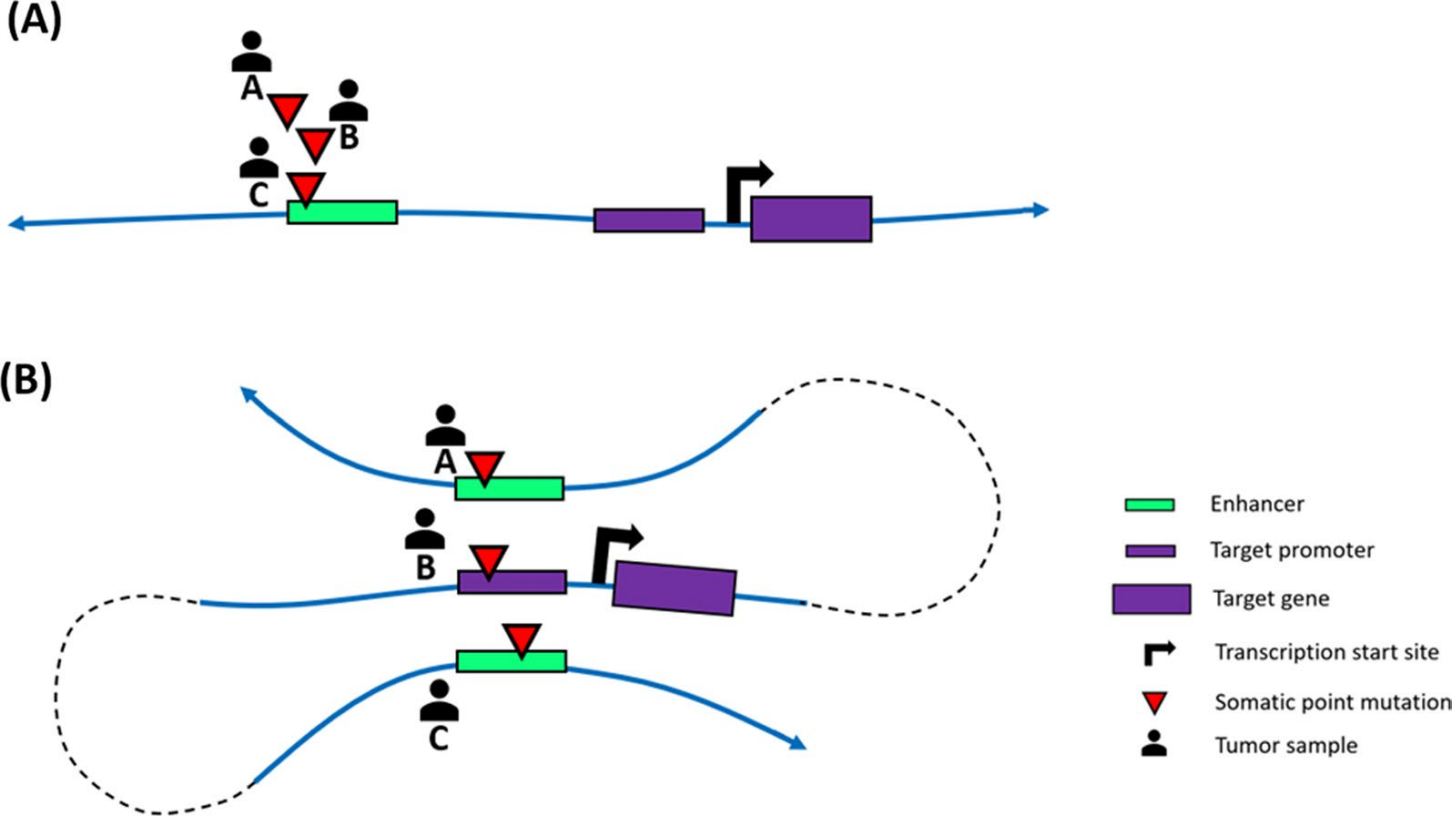
Illustration of two different recurrence model. **A** Site-level recurrence model: three point mutations from three individual tumor samples are clustered on the same site, making it a hotspot of somatic mutations. **B** Gene-level recurrence model: three point mutations from three individual tumor samples are scattered on three different regulatory elements, but when spatial conformation is taken into account, those mutations converge on the same target gene. Figure is adapted from [175]

Computational tools that detect non-coding somatic driver events contributing to tumor development have been developed [176–183]. These tools identify signs of positive selection by detecting enrichment of somatic mutations based on an estimated background mutation rate. In this sense, the PCAWG consortium remains the most comprehensive effort to identify non-coding driver events by employing multiple such tools to address the limitations of individual algorithms. However, one interesting finding from the PCAWG consortium was the continued scarcity of non-coding somatic mutational hotspots beyond the *TERT* promoter [44]. Although the presence of somatic drivers in regulatory elements is well accepted, their number is surprisingly low compared to the large numbers of non-coding mutations found in the typical tumor genome. This is partially due to the definition of what non-coding somatic mutations are deemed to be drivers. For a non-coding somatic mutation to be considered a driver, it must show evidence of positive selection (e.g., found to be recurrently mutated at a particular site; Fig. 6A). However, mutations at different sites may yield the same effect on an underlying functional unit. For example, driver genes are often mutated at different sites (exons) along their length [4], yet they drive tumorigenesis through affecting a common unit (a gene). Therefore, it remains possible that non-coding regulatory alterations driving tumorigenesis are more common than appreciated but scattered over the genome, thereby preventing the formation of highly recurrent hotspots at individual sites. Importantly, these non-coding mutations can still converge to specific genes or pathways, which makes them “recurrent” to those genes or pathways (Fig. 6B). Thus, cancer-driving regulatory mutations can be identified as recurrently targeting specific genes or pathways while not recurring at individual sites. Therefore, as with burden analysis for somatic eQTLs, mutations targeting genes that are on the same pathway are often collapsed to a single virtual locus.

Recent studies have incorporated chromosome conformation data to arrive on regulatory-gene connections within a regulatory recurrence network. For example, Sallari et al. [184] introduced the concept of a genetic “plexus” as a set of loci that are scattered over the linear genome but are located next to each other in the 3D nuclear space. These plexi were assembled using DNase-seq and histone modification ChIP-seq data to define genome-wide functional elements (e.g., enhancers) followed by the use chromatin interaction data (Hi-C) to identify their target genes. This allowed for the use of statistical tests to identify genome-wide driver genes with an excess of mutations in their plexi. This approach identified 15 candidate driver plexi in prostate cancer, including a plexus that converges on the *PLCB4* gene, which affects the PI3K cancer pathway [184]. Importantly, these non-coding mutations at driver plexi were not significant under the traditional recurrence test model. Using a similar “plexus” model, other studies have identified non-coding somatic mutations that converge on driver genes in breast cancer [42], lung cancer [175], prostate cancer [185] and ovarian cancer [186]. Further advancements in grouping-based statistical frameworks are expected to determine further important drivers of cancer development.

## Long-range interactions

Depending on the distance to the gene they regulate, eQTLs can be characterized as either *cis* or *trans*. Conventionally, eQTLs located within 1 Megabase (Mb) to a target gene’s transcription start site (TSS) are considered *cis*-eQTL, whereas those located > 1 Mb away (or between two chromosomes) are considered *trans*-eQTLs. Most enhancer-gene interactions identified are *cis*, as it is estimated that there is a median interaction distance of 120 kb between enhancer and target genes [187]. However, enhancers can act > 1 Mb away (*trans*) [75, 188]. Considerations in the 3C-based methodology (an exponential decrease in capture probability as genomic distance between two loci increases) make detecting ligation junctions between distant sites difficult but achievable such as was found in the physical association between the *MYC* locus and an oncogenic enhancer implicated in leukemia that acts 1.45 Mb away [189]. Therefore, while genome-wide identification of these loops using techniques such as Hi-C is promising, it will likely require enormous datasets and rigorous computational methods.

Similarly, the total number of reported long-range eQTLs (> 1 Mb) is relatively low [190]. As with Hi-C, the identification of longer-acting eQTLs presents additional challenges that complicate their identification. Unlike cis-eQTLs, where identification of target genes can be limited to certain genomic distances surrounding the loci of interest, trans-eQTL detection requires genome-wide testing. Importantly, testing all SNPs against all genes imposes a hefty multiple-testing burden, leading to only a small proportion of SNPs survive multiple testing corrections. Furthermore, the average effect size of *trans*-eQTLs is smaller [191], making detecting significant results more challenging.

**Fig. 7:**
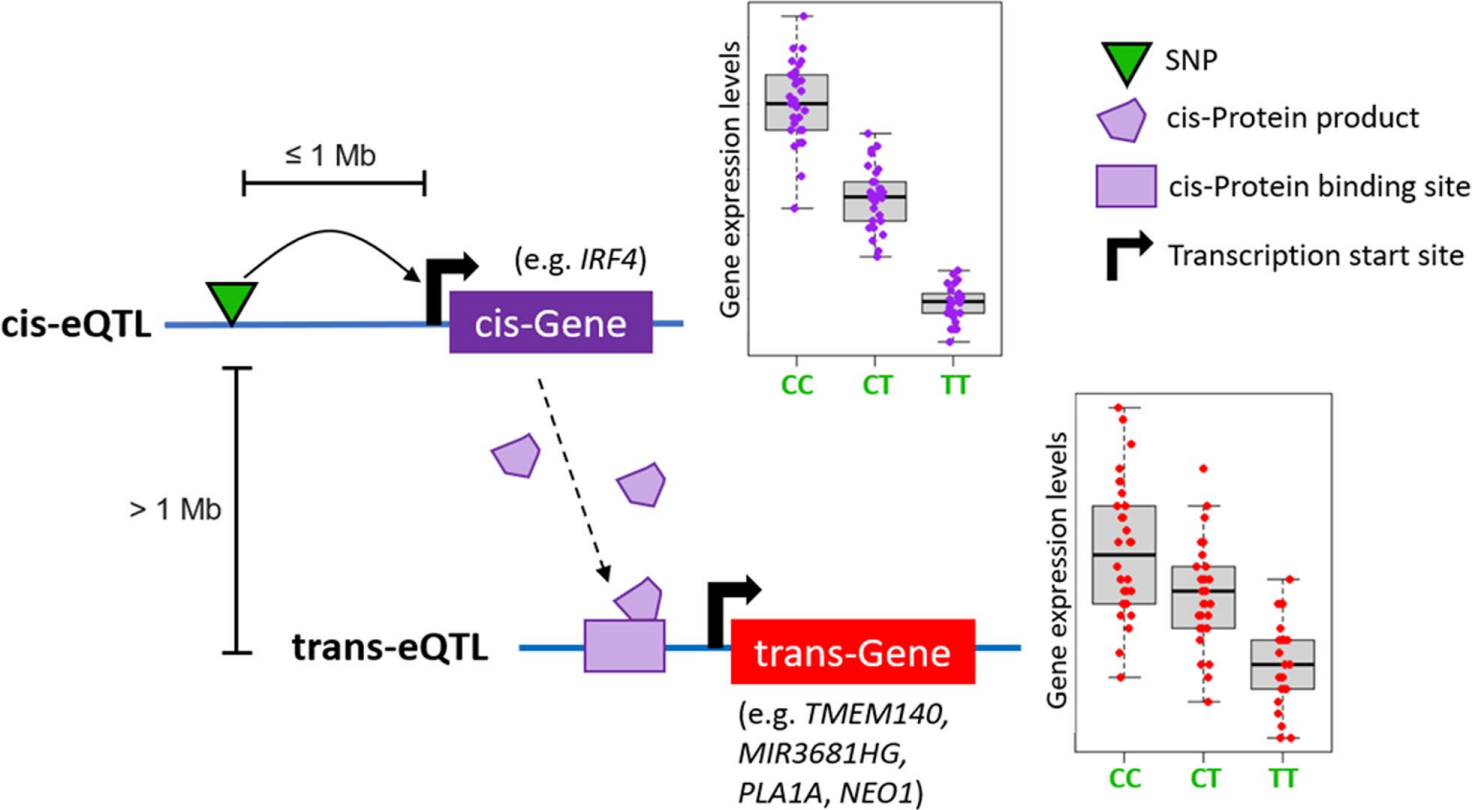
Many trans-eQTLs are also cis-eQTLs. A SNP may first alter the expression level of a *cis* diffusible mediator, such as a transcription factor, which then alters the expression of the *trans* genes through altering the binding of the transcription factor to its binding sites near the downstream *trans* genes

Several studies have successfully identified *trans*-eQTLs relevant to various cancers [192–195]. A recent analysis in melanocyte samples has identified rs12203592 (a SNP that was previously associated with human pigmentation phenotype [196]) as a genome-wide significant *trans*-eQTL that acts over 5 Mb away from its target genes [109]. Specifically, rs12203592 is found to target 4 *trans* genes (*TMEM140*, *MIR3681HG*, *PLA1A*, and *NEO1*). Interestingly, rs12203592 is also a *cis*-eQTL to the transcription factor IRF4. Thus, it is proposed that rs12203592 may indirectly affect the *trans* genes expression through its *cis* effect on IRF4. This suggests a melanocyte-specific *trans*-eQTL network regulated by the IRF4 transcription factor [109]. Many such *trans*-eQTLs are believed to affect the expression of a *cis* diffusible mediator (such as a transcription factor), which in turn affects the expression of the *trans* genes [197] (Fig. 7).

Given the large search space and statistical complexity, various approaches have been developed to improve the detection of *trans*-eQTLs. For example, by searching for SNPs with known *cis* associations [102], the search space for *trans* association is reduced, thereby reducing multiple-testing burden. Similarly, by searching for eQTLs with confirmed physical interactions (Hi-C) [91, 198], the detection of long-range interactions is improved. Other methods such as GMAC [199], CCmed [200], and others [201] regress the candidate *trans* genes on the *cis* genes to improve statistical power. Importantly, *trans*-eQTLs explain a substantial proportion of the underlying heritability of gene expression [202]. And *trans*-eQTLs are more likely to be tissue-specific modifiers of genes [203] and to target genes that are otherwise mutationally constrained [204]. Thus, despite their individually low effect sizes, *trans*-eQTLs are collectively crucial in explaining gene expression variability, which underlie differences in phenotype and disease susceptibility. Since it follows that many *trans*-eQTLs are not elucidated yet, further identification and analysis of these long-distance regulatory interactions are vital to complete our understanding of how cancers arise and develop.

## Conclusion and future outlook

The study of non-coding mutations requires the incorporation of multiple data types to better understand the key regulatory mechanisms disrupted by the mutations. Leveraging knowledge of enhancers and their connections to distant genes (eQTL and Hi-C) has helped in understanding the relationship between function, genome structure, and cancer. However, there are many improvements that can be made to existing studies.

Many methods have been proposed to solve the problem of mutational prioritization and gene target identification. However, as these methods sparsely agree with one another, it is important to better understand the underlying data being used and how to best incorporate this data to come to more accurate and synchronous conclusions.

The incorporation of accurate tissue- and cell-specific chromosome conformation and gene expression data will enhance the interpretation of non-coding mutations across all cancer types. This is especially relevant for the identification of *trans*-eQTLs, where cell-type heterogeneity has contributed to the low number of trans-eQTLs identified to date [203, 205]. Additionally, context specificity such as gene–environmental interactions will reveal chromatin loops and eQTLs specific to these environmental stimuli, identifying key changes in processes such as cell activation [206]. For example, future studies could use Hi-C and eQTL data from stimulated cells (e.g., UV-stimulated melanocytes) to interpret non-coding mutations that exert their effect upon specific environmental stimulation. Ultimately, these approaches will help us to develop personalized cancer treatments, targeted to impact the specific regulatory mechanisms altered by an individual’s specific mutational burden.

## Data Availability

Not applicable.
